# Investigating cognitive impairment, biopsychosocial barriers, and predictors of return to daily life among older stroke survivors

**DOI:** 10.3389/fneur.2024.1403567

**Published:** 2024-06-26

**Authors:** Alexandra Björck, Marie Matérne, Mialinn Arvidsson Lindvall, Gustav Jarl

**Affiliations:** ^1^School of Health Sciences, Faculty of Medicine and Health, Örebro University, Örebro, Sweden; ^2^University Health Care Research Center, Faculty of Medicine and Health, Örebro University, Örebro, Sweden; ^3^School of Behavioural, Social and Legal Sciences, Örebro University, Örebro, Sweden; ^4^School of Health, Care and Social Welfare, Mälardalen University, Västerås, Sweden; ^5^Department of Prosthetics and Orthotics, Faculty of Medicine and Health, Örebro University, Örebro, Sweden

**Keywords:** daily life, stroke, international classification of functioning disability and health (ICF), montréal cognitive assessment (MoCA), cognition

## Abstract

**Purpose:**

The aim was to investigate the associations between cognitive impairment and biopsychosocial factors among older stroke survivors and predictors of poststroke return to daily life.

**Materials and methods:**

This cross-sectional study involved 117 stroke survivors (61% men) with an average age of 77 years (range 65–91). The participants completed two questionnaires (Riksstroke and Short Form 36 questionnaires). The Montreal Cognitive Assessment (MoCA) was used to assess cognitive abilities. The International Classification of Functioning, Disability, and Health (ICF) framework guided the selection of biopsychosocial variables. We used Spearman’s correlation coefficient and multiple logistic regression in the analyses.

**Results:**

The average MoCA score was 21.7 points (range: 4–30, SD 5.6). The need for assistance from relatives and professionals, need for help with dressing and household chores, reliance on others for mobility, and reading and balance problems were correlated with more severe cognitive impairment (*r* = 0.20–0.33). Cognitive impairment, fatigue, and balance issues predicted an unfavorable return to daily life (odds ratio: 6.2–6.8).

**Conclusion:**

The study indicated that cognitive impairment is associated with difficulties in all ICF domains. Cognitive impairment, fatigue, and balance issues are associated with an unsuccessful return to daily life. Prioritizing these factors and screening for cognitive impairment with objective assessment tools may improve rehabilitation outcomes and enhance overall quality of life poststroke.

## Introduction

Stroke is a leading cause of long-term disability worldwide, impacting the lives of more than 100 million individuals (1, 2). Stroke disrupts cerebral blood flow, causing tissue damage and neurological deficits. The neuroplasticity enables the brain to adapt and reorganize post-stroke, aiding recovery (3) but a common consequence of stroke is poststroke cognitive impairment (PSCI). PSCI is a broad term that includes conditions ranging from mild cognitive impairment to dementia. Common disorders are problems with memory, attention, executive abilities, language, visual processing, and information management (4, 5). Usually, PSCI occurs within 3 months post-stroke, but can also develop over time (3). One year after stroke onset, one in four stroke survivors has PSCI (6, 7), and approximately seven of 10 stroke survivors will develop PSCI over time (8). PSCI often gives negative consequences on problem solving, organized planning and social interaction (9, 10), which has a negative effect on individuals’ overall health and well-being. There is a prioritized and ongoing global effort to understand the complexity of PSCI and develop interventions to prevent cognitive decline (11). PSCI predominantly affects the older population, which is growing globally (11, 12), making it a priority in stroke research (11). An emerging issue in stroke research is investigating how cognitive function is connected to other biopsychosocial factors to develop personalized interventions (4).

Previous research has indicated that PSCI affects several biopsychosocial aspects. It affects the overall recovery process poststroke due to the negative impact on the body’s motor function recovery (13). PSCI makes it more challenging to participate in physical rehabilitation; therefore, persons with PSCI are less physically active (14). Individuals with PSCI are also more prone to psychological deficits such as depression and fatigue (15, 16), which further affects their ability to engage in various leisure and social activities (17). PSCI affects both basic and instrumental activities of daily living, such as dressing, performing personal hygiene and household chores, and cooking (18). It also impairs communication (19), participation in social activities and interactions with others (18, 20). Despite extensive PSCI research, the understanding of how different levels of PSCI affect biopsychosocial factors in older stroke survivors is incomplete. There is also limited research exploring which factors are associated with a successful return to daily life for older persons with PSCI. Previous studies investigating factors associated with return to daily life and leisure activities after stroke have shown that cognitive ability, age, and mobility are important factors (21, 22). However, most related studies have focused on younger stroke survivors (23, 24).

This study adopted a biopsychosocial perspective and used the *International Classification of Functioning, Disability and Health (ICF)* model (25) as a framework. This approach was chosen to capture the complexity of the interaction between PSCI and daily life. The study aimed to investigate (1) the associations between levels of cognitive impairment and biopsychosocial factors and (2) how these factors are associated with return to daily life. Through comprehensive insights into these biopsychosocial factors, poststroke well-being and recovery can be enhanced, allowing for tailored interventions for affected individuals.

## Methods

### Design

This was a cross-sectional study with data from a stroke population in a medium-sized Swedish municipality. This study was part of the Kumla Stroke Study and received ethical approval from the Swedish Ethical Review Authority (Reference No. 2019-02359).

### Participants

**Figure 1:**
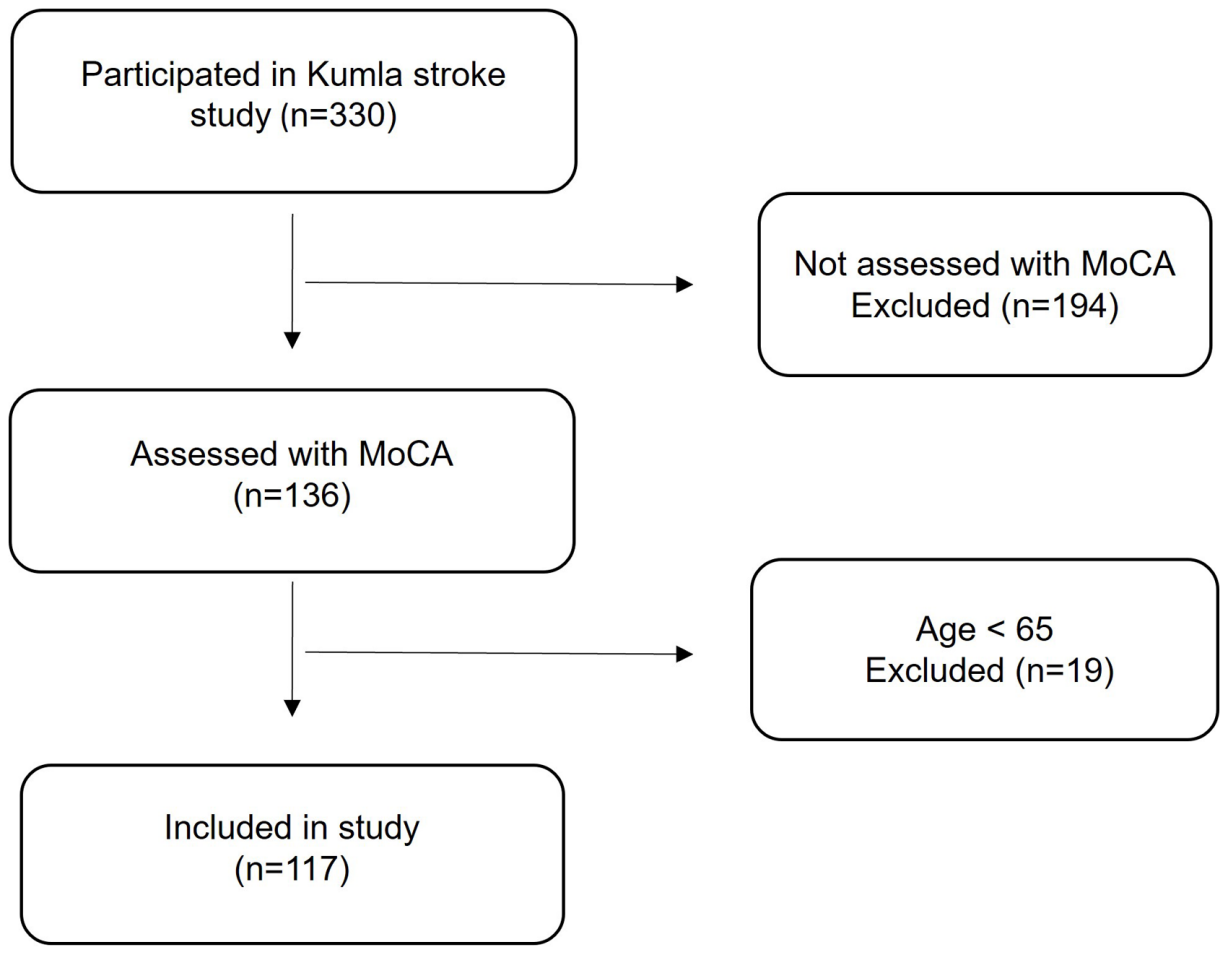
Flowchart of participants included in the study.

In the Kumla Stroke Study, a total of 330 people diagnosed with stroke and living in the Kumla municipality were identified as of December 31, 2019 (26). The Swedish Stroke Register (Riksstroke), which is a nationwide quality registry for stroke care (27), medical journals and the local health care center diagnostic register (28), were used to identify the participants. From these 330 persons, we included a subsample who (1) had completed the Montreal Cognitive Assessment (MoCA) (29) and (2) were ≥ 65 years old (Figure 1).

### Framework, measurement tools, and questionnaires

All the data were collected between October 2019 and June 2020. The Riksstroke (27) and Short-Form Health Survey (SF-36) (30) questionnaires were sent by mail and were completed by the participants themselves or with help from a family member. Participants could if they requested also receive support in completing the questionnaires by two physiotherapists, which also conducted the MoCA assessments, either at the local health care center or in the participant’s home. All questionnaires and assessment tools, which were used in the study, were the Swedish versions.

*The International Classification of Functioning, Disability and Health* (ICF) (25) is a biopsychosocial model from the World Health Organization (WHO). The ICF describes a person’s body functions, body structures, activity, participation, and contextual factors and is used as a framework to describe a person’s overall status of health and well-being. The variables used in this study were selected from the Riksstroke and SF-36 questionnaires to cover a wide range of biopsychosocial factors, which were mapped to ICF codes. ICF mapping rules were used (31) (Table 1).

**Table 1:** Description of ICF domains, categories and codes of the Riksstroke and SF-36 questionnaires.

ICF domains	ICF categories	ICF codes	Questionnaires	Questions
**Body functions**	-	-	-	-
Mental functions	Energy and drive functions	b130	Riksstroke	Do you feel tired?
	Memory functions	B144	Riksstroke	Do you have memory problems?
	Emotional functions	b152	Riksstroke	Do you feel depressed?
Sensory functions and pain	Sensation of pain	b280	Riksstroke	Do you feel pain?
Neuromusculoskeletal and movement related functions	Control of voluntary movement functions	b760	Riksstroke	Do you have problems with balance?
**Activities and participation**				
Learning and applying knowledge	Reading	d166	Riksstroke	Do you have problems with reading?
Communication	Speaking	d330	Riksstroke	Do you have problems with speech?
Mobility	Moving around in different locations	d460	Riksstroke	Do you have problems with mobility?
Self care	Dressing	d540	Riksstroke	Do you need dressing assistance?
Domestic life	Doing housework	d640	Riksstroke	Do you need household chore assistance?
Interpersonal interactions and relationships	Informal social relationships	d750	SF36	Social functioning domain
	Family relationships	d760		
**Contextual factors**				
Environmental factors				
Support and relationships	Immediate family	e310	Riksstroke	Do you live alone?
	Friends	e320	Riksstroke	Do you need help from relatives?
	Personal care providers and personal assistance	e340	Riksstroke	Do you need professional assistance?
Personal factors				
Age (65–74, 75–79, and 80–91 years)			Riksstroke	
Sex (female, male)			Riksstroke	
General question regarding activities and participation			Riksstroke	Have you been able to return to the life and activities that you had before your stroke?

*The Montreal Cognitive Assessment* (MoCA) (29) is a screening instrument for cognitive impairment and has been validated for use in stroke survivors. The instrument evaluates attention, concentration, executive functions, memory, language ability, visuoconstructive ability, abstract thinking, numeracy, and orientation. The highest possible score is 30 points. Based on previous studies, the scores were categorized as follows: no cognitive impairment (24–30 points) (32), mild cognitive impairment (18–23 points), and severe cognitive impairment (1–17 points) (33).

*The Riksstroke 1-year follow-up questionnaire* is a Swedish questionnaire that has been validated for use in stroke survivors (27, 34). It consists of 46 questions regarding stroke survivors’ life situation, daily activities, and need for support and assistance. This study used 15 questions mainly concerning physical, psychological, and activity functions (Table 1). Two questions were converted to trichotomous variables *(Do you feel tired? Do you feel depressed? Do you feel pain?)* and two questions were converted to dichotomous variables (*Do you need household assistance? Have you been able to return to the life and activities that you had before your stroke?*).

*The Short Form Health Survey 36* (SF-36) is a validated quality of life questionnaire consisting of 36 questions divided into eight domains (30). This study used the Social Function domain (SF) to describe a person’s ability to socialize; the SF domain consists of two questions: (I) *During the past 4 weeks, to what extent has your physical health or emotional problems interfered with your usual interactions?* and (2) *During the last 4 weeks, how much of the time has your physical health or emotional problems interfered with your ability to socialize?* (Table 1).

### Statistical analysis

All analyses were conducted by using IBM SPSS statistics version 27 (IBM Corp., Armonk, NY, United States). Descriptive statistics describing the characteristics of the participants are presented as the mean, minimum, maximum, and standard deviation (SD). Spearman’s rank correlation coefficient (Rho) was used to analyze correlations between the independent variables in Table 1 and the dependent variable (cognitive impairment). Cohen’s levels of correlation were used (35).

Logistic regression was used to investigate predictors of return to daily life poststroke. The answer to the question “*Have you been able to return to the life and activities that you had before your stroke?*” was used as the dependent variable. Univariate logistic regression analysis was used to determine which independent variables should be included in the multiple regression analysis. A *p* value <0.05 was regarded as statistically significant. A test for multicollinearity with all the independent variables was conducted before the variables were included in the multiple regression analysis, and no substantial collinearity was found (tolerance >0.66 and variance inflation factor < 1.52).

## Results

The study sample consisted of 117 persons (61% men), and the average age was 77 years, with a range from 65 to 91 years (SD 6.1). Over half of the participants resided with a spouse, and none were living in care homes; all participants were living in regular housing (Table 2).

**Table 2:** Results from Riksstroke and SF-36 questionnaires divided into groups based on MoCA scores.

		Level of cognitive impairment according to the MoCA score, described in %					
		None	Moderate	Severe	Total	Spearman’s	
*Variables*		24–30 points	18–23 points	1–17 points		Rho	*p* value
ICF-Body function (*n* = 114–116)							
Feel tired	Never/rarely	21	5	15	16	0.11	0.245
	Sometime	46	56	44	49		
	Often/constantly	32	38	41	36		
Feel depressed	Never/rarely	58	41	32	47	0.24	0.090
	Sometime	35	38	48	39		
	Often/constantly	7	22	20	14		
Problems with balance	Yes	30	58	48	42	0.20	0.037
	No	70	42	52	58		
Feel pain	Never/rarely	32	34	37	34	0.09	0.346
	Sometime	38	34	48	39		
	Often/constantly	30	31	15	27		
Problems with memory	Yes	20	27	44	28	0.21	0.025
	No	80	73	56	72		
ICF-Activity/participation (*n* = 111–117)							
Problems with reading	Yes	4	9	19	9	0.21	0.027
	No	96	91	81	91		
Problems with speech	Yes	11	24	19	16	0.12	0.202
	No	89	76	81	84		
Problems with mobility	Move independent in-and outdoors	96	88	82	91	0.21	0.024
	Move independent indoors, not outdoors/dependent in-and outdoors	4	12	19	10		
Need dressing assistance	Yes	0	15	22	9	0.33	<0.001
	No	100	85	78	91		
Need household chore assistance	Yes	26	33	52	34	0.20	<0.001
	No	74	67	48	66		
Problems with socializing	None/some of the time	73	58	48	64	0.15	0.133
	Sometime	7	19	43	18		
	Most of the time/all of the time	20	23	10	19		
ICF-Contextual factors (*n* = 113–117)							
Need professional assistance	Yes	4	12	19	9	0.21	0.022
	No	96	88	81	91		
Live alone	Yes	33	52	41	40	0.10	0.280
	No	67	48	59	60		
Need help from close relatives	Yes, some part	25	31	36	29	0.31	<0.001
	Yes, dependent	2	13	24	10		
	No	73	56	40	61		
Sex	Female	40	39	37	39	0.02	0.787
	Male	60	61	63	61		
Age groups	65–74 years	44	24	26	34	0.18	0.056
	75–79 years	32	30	41	33		
	80–91 years	25	46	33	33		

The average MoCA score was 21.7 points, with a range from 4 to 30 points (SD 5.6). The results of the MoCA indicated that nearly half of the participants (48.7%) had no cognitive impairment, more than a fourth of the participants (28.2%) had moderate cognitive impairment, and less than a fourth of the participants had severe cognitive impairment (23.1%). In terms of self-reported memory problems, 20% of the individuals without cognitive impairment reported having memory problems. In contrast, among the participants with moderate cognitive impairment, fewer than one-third reported memory problems, and among those with severe cognitive impairment, less than half reported memory issues. More than 80% of the participants experienced fatigue, and more than half-experienced pain and depression. Forty percent of the participants also had problems with balance and socializing. The need for assistance with household chores was the most common assistance need, and most of the assistance was provided by close relatives (Table 2).

### Associations between cognitive impairment and biopsychosocial factors

Severe cognitive impairment was significantly associated with more difficulties in eight out of 16 biopsychosocial factors: *dressing assistance, assistance from close relatives, assistance from professionals, reliance on others for mobility, household chore assistance, reading, balance*, *and memory problems* (Table 2). All eight variables had positive correlations (Rho = 0.02–0.33, weak-moderate); that is, more severe cognitive impairment was associated with more difficulties in these areas. However, the association between cognitive level and balance was not linear; participants who had moderate PSCI had more problems with balance than did those with no PSCI or severe PSCI.

### Associations between biopsychosocial factors and return to daily life

The univariate regression results (Table 3) showed that the biopsychosocial factors *household chore assistance, fatigue, balance, cognitive impairment, speech, dressing assistance, reading*, *and mobility* were significantly associated with *return to daily life and activities* (*p* ≤ 0.001–0.04).

**Table 3:** Univariate and multiple logistic regression analysis.

	Univariate regression		Multiple regression		
Independent variables	Odds ratio	*p* value	Odds ratio	Returned to everyday life (in %)	*p* value
Sex (female)	0.67	0.31			
Age groups		0.98			
65–74 years (ref)					
75–79 years	0.96				
80–91 years	1.1				
Do you live alone? (no)	1.4	0.43			
Do you feel tired?		<0.001			
Never/rarely (ref)				72	0.012
Sometimes	2.8		1.5	48	0.54
Often/always	12.6		6.8	17	0.011
Do you have problems with balance? (no)	8.5	<0.001	6.2	15	<0.001
Cognitive impairment		0.003			
No impairment 24–30 points (ref)				54	0.014
Moderate impairment 18–23 points	2.5		2.1	32	0.21
Severe impairment 1–17 points	5.2		6.4	18	0.004
Do you have problems with speech? (no)	4.4	0.012			
Do you need dressing assistance? (no)	7.6	0.016			
Do you have problems with reeding? (no)	6.9	0.026			
Do you have problems with mobility?		0.015			
Move independent in-and outdoors (ref)					
Move independent indoors, not outdoors/	7.8				
Dependent in-and outdoors					
Do you feel depressed?		0.060			
Never/rarely (ref)					
Sometimes	2.6				
Often/always	2.4				
Do you have problems with socializing? (SF dimension)	0.99	0.11			
Do you feel pain?		0.41			
Never/rarely (ref)					
Sometimes	1.5				
Often/always	1.9				

Dependent variable: Have you been able to return to the life and activities that you had before your stroke?

According to our multiple regression analysis (Table 3), *cognitive impairment*, *balance*, *and fatigue* were identified as significant predictors of nonreturn to daily life for stroke survivors. The odds of returning to daily life and activities were 6.2–6.8 times greater for persons who never or rarely felt tired, had no problems with balance and had no cognitive impairment than for those who had problems in these areas. Less than 20% of the participants who had problems with balance, often or always felt tired or had severe cognitive impairment returned to daily life and activities after stroke.

## Discussion

The overall findings of this study indicate significant associations between cognitive impairment and various biopsychosocial factors across all ICF domains. While the weak to moderate correlations indicate that the relationships between these factors are not notably strong, this study’s findings still emphasize the importance of recognizing cognitive impairment in stroke care but also the need for early detection, considering its impact on stroke survivors’ return to daily life.

One of the aims of this study was to investigate how cognitive impairment was associated with biopsychosocial factors; severe cognitive impairment was most strongly associated with factors within the “*Activity and participation*” and “*Contextual factors*” ICF domains. The findings revealed that an increased need for assistance in tasks involving dressing, mobility, and household chores was associated with more severe cognitive impairment. This finding is in line with that of another study highlighting the association between an increased need for support in basic and instrumental activities of daily living and more severe cognitive impairment (36). Severe cognitive impairment was also associated with an increased demand for assistance from both family members and professionals. Earlier findings indicated that people with cognitive impairment required 2–5 times more support than did those without cognitive impairment (37). Our findings imply that cognitive impairment impacts a wide range of daily activities. By combining the ICF model with the MoCA, as demonstrated in this study, we were able to pinpoint the specific biopsychosocial factors most impacted by the severity of cognitive impairment. Understanding individual needs can facilitate the customization of stroke rehabilitation programs and enhance stroke survivors’ daily lives, as well as ease the burden on families and society.

The second aim of this study was to explore the associations between biopsychosocial factors and return to daily life. Return to daily life falls under the ICF—domain of “*Activity and participation*.” Return to daily life was associated with three factors: cognitive impairment, balance, and fatigue. These factors were all included in the ICF domain of “*Body functions*.” The first factor associated with an unsuccessful return to daily life was cognitive impairment, which often affects stroke survivors’ executive functions and has a negative impact on problem solving, planning, and social interaction, which are all essential factors for returning to daily life (9, 10). However, a similar study exploring predictors of return to daily activities poststroke found no associations with cognitive impairment (21). However, that study relied on participants’ self-assessments of their cognitive abilities, which may have influenced the results. In our study, using the MoCA, we revealed a significant difference between participants’ self-perceived memory problems and their actual MoCA results. Less than one-third of the participants with moderate cognitive impairment and less than half of the participants with severe cognitive impairment recognized their memory issues. If individuals fail to acknowledge cognitive problems, it becomes crucial for health care providers to step in. Previous research frequently highlights the tendency to overlook cognitive impairment poststroke in favor of physical concerns (4, 38). For instance, a study showed that 70% of stroke survivors discharged with clinical successful recovery according to modified Rankin Scale, and no apparent functional disability, yet demonstrated various cognitive deficits 3 months post-stroke (39). Studies also indicate that health care professionals struggle to detect and prioritize cognitive impairment within brief care periods (4, 5). In essence, recognizing the gap between self-perceptions and objective assessments underscores the urgent need for comprehensive cognitive evaluation in clinical stroke care practice but also the need to do cognitive assessment follow-ups.

Balance challenges were also significantly associated with an unsuccessful return to daily life. Another study revealed that the primary hindrance to resuming physical activity and daily activities was fear of falling due to balance issues (39). Moreover, avoiding situations perceived as posing a fall risk has emerged as the primary cause of the decrease in physical mobility, limited social participation, and reduced quality of life among stroke survivors (40, 41). This underscores the importance of considering balance challenges in a broader context and recognizing the wider psychosocial impact. Using a biopsychosocial approach and combining interventions that provide support regarding both balance and psychological issues may therefore enhance overall recovery for stroke survivors and improve their ability to return to daily life.

In this study, experiencing fatigue emerged as the final factor associated with an unsuccessful return to daily life. A total of 85% of the participants experienced fatigue poststroke, which aligns with previous findings (42). Poststroke fatigue notably affects quality of life and limits individuals’ participation in various daily activities (43). Research has also indicated that stroke survivors with both reduced physical abilities and fatigue have more difficulties returning to previous leisure activities (22). Promoting physical activity, particularly aerobic exercise, is a promising treatment for reducing poststroke fatigue (42). Furthermore, this form of physical activity has also been shown to be effective at preventing or delaying cognitive decline (44, 45); therefore, incorporating this approach in stroke rehabilitation may be beneficial.

We found no association between older age and more severe cognitive impairment or an unfavorable return to daily life. The results of this study contradict those of several other studies that highlight that older age is a strong risk factor for poststroke cognitive impairment (12, 46, 47) and a more challenging return to daily life (21). An explanation for the contradictory result may be that this study focused on stroke survivors aged 65 years and older, which aligns with the retirement age in Sweden. The narrow age range, from 65 to 91 years, might not have been broad enough to impact the results. Alternatively, people with PSCI older than 65 years of age may face similar challenges, regardless of their specific age.

In summary, this study highlights the complex dynamics between cognitive impairment and various biopsychosocial factors impacting daily life poststroke. These findings highlight the importance of screening for cognitive impairment to customize stroke rehabilitation programs and incorporate a broader biopsychosocial perspective in rehabilitation programs.

### Limitations and strengths

When interpreting these study results, it is important to consider aspects that might have impacted the study’s outcomes. The lack of homogeneity in stroke severity and years lived with stroke among the participants could have influenced the findings. For example, the participants might have experienced difficulties recalling their return to daily life poststroke, particularly if the stroke had occurred several years ago. The participants had various levels of cognitive impairment, which may affect their ability to answer questions about how the stroke impacted their biopsychosocial and activity performance, especially for those with severe impairment. To ensure the accuracy in the responses, participants could receive support from a family member or a trained research assistant when completing the questionnaires. However, it is important to acknowledge that the participant’s answers are self-reported and should be interpreted considering their cognitive impairment. Furthermore, a cross-sectional study provides only a snapshot of stroke survivors’ lives at a specific point in time. This study may provide a plausible picture of the comprehensive challenges faced by older stroke survivors coping with cognitive impairment today. A notable strength of this study was the use of an objective cognitive assessment tool. This decision ensured a more accurate and reliable evaluation of cognitive impairment, strengthening the robustness of our study’s findings. Additionally, by using the ICF framework, this study comprehensively analyzed a spectrum of biopsychosocial factors, offering a broad perspective on poststroke cognitive impairment. The use of the ICF framework may also facilitate future valuable comparisons with other studies that share a similar analytical framework.

### Conclusion

Our findings indicate that cognitive impairment is associated with all ICF domains, and that cognitive impairment, fatigue, and balance predict an unfavorable return to daily life and activities. Notably, we identified a higher prevalence of cognitive impairment than what individuals themselves perceived, highlighting the critical need to prioritize its detection by using objective assessment tools. These findings may provide valuable insights for clinical practice by underscoring the importance of addressing a wide range of biopsychosocial factors associated with cognitive impairment for older stroke survivors understanding these factors can help healthcare professional prioritize interventions and improve rehabilitation outcomes. Furthermore, our research emphasizes the necessity of regular screening for cognitive impairment, allowing for tailored interventions to meet each individual’s specific needs.

## Data availability statement

The raw data supporting the conclusions of this article will be made available by the authors, without undue reservation.

## Ethics statement

The studies involving humans were approved by Swedish Ethical Review Authority. The studies were conducted in accordance with the local legislation and institutional requirements. The participants provided their written informed consent to participate in this study.

## Author contributions

AB: Writing – original draft, Writing – review & editing. MM: Writing – original draft, Writing – review & editing. MA: Writing – original draft, Writing – review & editing. GJ: Writing – original draft, Writing – review & editing.
